# Curcumin Enhances the Effect of Chemotherapy against Colorectal Cancer Cells by Inhibition of NF-κB and Src Protein Kinase Signaling Pathways

**DOI:** 10.1371/journal.pone.0057218

**Published:** 2013-02-22

**Authors:** Mehdi Shakibaei, Ali Mobasheri, Cora Lueders, Franziska Busch, Paviz Shayan, Ajay Goel

**Affiliations:** 1 Institute of Anatomy, Ludwig-Maximilian-University Munich, Munich, Germany; 2 Division of Veterinary Medicine, School of Veterinary Medicine and Science, Faculty of Medicine and Health Sciences, University of Nottingham, Sutton Bonington Campus, Sutton Bonington, United Kingdom; 3 Department of Thoracic and Cardiovascular Surgery, Laboratory for Tissue Engineering, German Heart Institute Berlin, Berlin, Germany; 4 Investigating Institute of Molecular Biological System Transfer, Tehran, Iran; 5 Gastrointestinal Cancer Research Laboratory, Division of Gastroenterology, Baylor Research Institute and Charles A. Sammons Cancer Center, Baylor University Medical Center, Dallas, Texas, United States of America; The University of Texas M. D. Anderson Cancer Center, United States of America

## Abstract

**Objective:**

Development of treatment resistance and adverse toxicity associated with classical chemotherapeutic agents highlights the need for safer and effective therapeutic approaches. Herein, we examined the effectiveness of a combination treatment regimen of 5-fluorouracil (5-FU) and curcumin in colorectal cancer (CRC) cells.

**Methods:**

Wild type HCT116 cells and HCT116+ch3 cells (complemented with chromosome 3) were treated with curcumin and 5-FU in a time- and dose-dependent manner and evaluated by cell proliferation assays, DAPI staining, transmission electron microscopy, cell cycle analysis and immunoblotting for key signaling proteins.

**Results:**

The individual IC_50_ of curcumin and 5-FU were approximately 20 µM and 5 µM in HCT116 cells and 5 µM and 1 µM in HCT116+ch3 cells, respectively (*p<0.05*). Pretreatment with curcumin significantly reduced survival in both cells; HCT116+ch3 cells were considerably more sensitive to treatment with curcumin and/or 5-FU than wild-type HCT116 cells. The IC_50_ values for combination treatment were approximately 5 µM and 1 µM in HCT116 and 5 µM and 0.1 µM in HCT116+ch3, respectively (*p<0.05*). Curcumin induced apoptosis in both cells by inducing mitochondrial degeneration and cytochrome c release. Cell cycle analysis revealed that the anti-proliferative effect of curcumin and/or 5-FU was preceded by accumulation of CRC cells in the S cell cycle phase and induction of apoptosis. Curcumin potentiated 5-FU-induced expression or cleavage of pro-apoptotic proteins (caspase-8, -9, -3, PARP and Bax), and down-regulated anti-apoptotic (Bcl-xL) and proliferative (cyclin D1) proteins. Although 5-FU activated NF-κB/PI-3K/Src pathway in CRC cells, this was down-regulated by curcumin treatment through inhibition of IκBα kinase activation and IκBα phosphorylation.

**Conclusions:**

Combining curcumin with conventional chemotherapeutic agents such as 5-FU could provide more effective treatment strategies against chemoresistant colon cancer cells. The mechanisms involved may be mediated via NF-κB/PI-3K/Src pathways and NF-κB regulated gene products.

## Introduction

Colorectal cancer (CRC) is one of the leading causes of death in men and women, and ranks among the third most common cancers globally [1]. The prevalence of CRC is still increasing despite our enhanced understanding of the pathogenesis of this disease, as well as establishment of improved screening strategies for this malignancy. It has been reported that almost 50% of the patients with CRC, will develop recurrent disease, indicating that currently available treatment regimens are not able to control this deadly disease and there is an imperative need for improved therapies [2]. 5-Fluorouracil (5-FU) is one of the classical drugs used as chemotherapeutic agent against CRC. 5-FU treatment suppresses tumor cell growth and induces apoptosis by incorporation of its metabolites into DNA and RNA through thymidylate synthase. However, gains made by the chemotherapeutic efficacy of 5-FU are somewhat limited in patients with colorectal cancer, primarily due to acquired progressive resistance of CRC cells to 5-FU and toxicity to surrounding healthy cells [3], [4].

Most tumors activate the transcription factor nuclear factor-κB (NF-κB), whereas natural chemopreventive agents suppress it, indicating a strong link between tumor biology and the anti-cancer effects of various natural compounds [5]. In contrast to healthy cells, in the majority of solid and hematopoietic tumor cell lines NF-κB is constitutively active [6]. Furthermore, pro-inflammatory cytokines, chemotherapeutic agents and radiation therapy, that induce apoptosis also activate NF-κB [7] and thus may mediate chemoresistance and radioresistance of tumor cells [8]. Interestingly, inhibition of NF-κB in tumor cells blocks proliferation, causes cell cycle arrest, and leads to apoptosis, suggesting a central role for this transcription factor in cell proliferation and survival [9]. NF-κB plays an important role in cell proliferation and malignant transformation in different cells, binding to DNA target sites as homo- or heterodimer to influence downstream gene expression [10], [11].

CRC is believed to occur as a consequence of stepwise accumulation of genetic alterations in various genes leading to enhanced genomic instability. Such alterations have direct influence on metastasis-associated genes, oncogenes and tumor suppressor genes [12], [13]. Furthermore, there is increased recognition that besides genetic events in CRC, alterations in gene expression may be mediated by epigenetic alterations including aberrant methylation of DNA, histone modifications and chromatin remodeling [14], [15]. Additionally, the mismatch repair (MMR) system plays an essential role in proofreading DNA synthesis errors during cell replication. Damage to the MMR system causes genetic instability, leading to alterations in the cell phenotype, rendering the cell more susceptible to neoplastic transformation and facilitating the development of chemotherapeutic resistance. DNA MMR proteins, such as hMSH2, hMSH3, hMSH6, hMLH1, hPMS2 and hMLH3 play an important role in both sporadic and familial varieties of human CRC [16], [17], [18], [19]. In fact, restoration of the MMR defect by re-expression of hMLH1or hMSH2 by chromosome transfer confers increased sensitivity to the agents [20], [21].

CRC is a preventable disease, and is heavily influenced by environmental, lifestyle and dietary factors. In this context, there is a growing body of literature suggesting that several natural occurring substances as dietary supplements can reduce cancer risk and have been reported to hold a central role in the development of anti-tumor drugs [22], [23]. Natural products with the ability to inhibit activation of the nuclear transcription factor NF-κB could have therapeutic potential against tumor development like CRC. Curcumin (diferuloylmethane) is the biologically active phytochemical component present in the spice turmeric (*Curcuma longa*), and has been shown to be a potent inhibitor of NF-κB activation in several cell types at nontoxic concentrations in humans [24]. The anti-tumor property of curcumin, is partly due to the arrest of cancer cells in S, G2/M cell cycle phase and the induction of apoptosis. Furthermore, curcumin inhibits the growth of DNA MMR-deficient colon cancer cells [25], [26]. It has also been reported that curcumin down-regulates constitutively activated kinase PI-3K/AKT pathways in T cell leukemia cells, which suppresses proliferation and induces caspase-dependent apoptosis [27].

Given that colorectal cancer patients with DNA mismatch repair deficiency do not benefit from 5-FU based chemotherapy, we used a pair of isogenic cell lines, HCT116 (MMR-deficient, due to hypermethylation of MLH1 gene) and HCT116+ch3 (MMR-proficient, due to stable transfer of chromosome 3 bearing wild type copy of the MLH1 gene). The aim of this study was to examine the chemosensitization potential of curcumin in 5-FU-based chemotherapy in MMR-deficient and -proficient CRC cells.

## Materials and Methods

### Antibodies

Antibodies against MMP-9 (MAB 911) and active caspase-3 (AF835) were obtained from R&D Systems, Inc., (Heidelberg, Germany). Monoclonal anti-PARP [poly(ADP-ribose)polymerase] (7D3-6) antibodies were purchased from Becton Dickinson (Heidelberg, Germany). Cyclo-oxygenase-2 (160–112) antibody was obtained from Cayman Chemical (Ann Arbor, MI, USA). Antibodies to β-actin (A5316) were from Sigma (Munich, Germany). Antibodies to Bax were obtained from Santa Cruz Biotechnology (Santa Cruz, CA). Antibodies against phospho-specific IκBα (Ser-32/36), p65 and phospho-specific p65 (Ser536) were obtained from Cell Technology (Beverly, MA). Anti-IκB kinase (anti-IKK)-α and (anti-IKK)-β antibodies were obtained from Imgenex (Hamburg, Germany). Alkaline phosphatase linked sheep anti-mouse and sheep anti-rabbit secondary antibodies for immunoblotting were purchased from Millipore (Schwalbach, Germany). All antibodies were used at concentrations and dilutions recommended by the manufacturer (dilutions ranged from 1:100 for immunomorphological experiments to 1:10,000 for Western blot analysis).

### Growth Media, Chemicals, and Cytokines

Growth medium (Ham's F-12/Dulbecco's modified Eagle's medium (50:50) containing 10% fetal calf serum (FCS), 25 µg/ml ascorbic acid, 50 IU/ml streptomycin, 50 IU/ml penicillin, 2.5 µg/ml amphotericin B, essential amino acids and L-glutamine) was obtained from Seromed (Munich, Germany). Trypsin/EDTA (EC 3.4.21.4) was purchased from Sigma. Epon was obtained from Plano (Marburg, Germany). 5-FU was purchased from Sigma (Munich, Germany). Curcumin with a purity of greater than 95% was purchased from Indsaff (Punjab, India). This commercial source of curcumin contains three major components: Diferuloylmethane (the most abundant and active component of turmeric) (82%) and its derivatives demethoxycurcumin (15%) and bisdemethoxycurcumin (3%), together referred to as curcuminoids [24], [28]. Curcumin was dissolved in dimethylsulfoxide (DMSO) as a stock concentration of 5000 µM and stored at −80 °C. Serial dilutions were prepared in culture medium. A 100 mM stock of 5-FU was prepared in absolute DMSO and stored at −20 °C. For treatment, the 5-FU stock solution was diluted in DMEM/F12 and added to cultures to achieve the desired concentration. The final concentration of DMSO was less than 1% of drug treatment. After culturing the cells to 70–80% confluency, they were treated with 5-FU, curcumin or their combination (in each case of the combination treatment, the cells were first pretreated with curcumin for 12 h or the indicated times and then exposed to 5-FU for 24 h or the indicated times).

### Cell lines and cell culture

Human colon cancer cells (HCT116 wild type) were obtained from Sigma-Aldrich (Munich, Germany). HCT116+ch3, which was made MLH1-proficient by the stable transfection of chromosome 3 bearing a wild-type copy of the *hMLH1* gene were prepared as originally described [21]. The HCT116 and HCT116+ch3 cells were used to investigate the efficacy of combined therapy of 5-FU and curcumin. The cells were maintained in tissue culture flasks in DMEM/F12 (4.5 g/L D-glucose) supplemented with 10% FBS and 1% antibiotic/antimycotic in a humidified incubator at 37 °C in an atmosphere of 95% air and 5% CO2. The medium was changed every three days, and cells were passaged using trypsin/EDTA.

### Cell proliferation assay

The effect of 5-FU, curcumin and their combination on proliferation and viability of HCT116 and HCT116+ch3 cells was determined by the 3-(4,5-dimethylthiazol-2-yl)-2,5-diphenyltetrazolium bromide (MTT) uptake method as described previously [29]. Briefly, the cells (2,500 per well) were exposed to different concentrations of 5-FU or curcumin, each in triplicate, in a 96-well plate for the indicated time periods at 37 °C to determine the individual IC_50_ values (50% cell growth inhibitory concentrations). Additionally, in another set of experiments, cells were pretreated with 5 µM curcumin for 4 h and then co-treated with different concentrations of 5-FU (0, 0.1, 1, 2, 3, 4 and 5 µM) for 24 h to determine optimum dose for the combination treatment. MTT solution (5 mg/ml) was added to each well and the plate was incubated for 2 h at 37 °C. The lysis buffer (20% SDS and 50% dimethyl formamide) was added, and the cells were further incubated overnight at 37 °C. The absorbance of the cell suspension was measured at 570 nm using a microplate reader Revelation 96-well multiscanner (Dynex Technologies, Chantilly, VA). The data obtained were calculated and represented as percentage survival with respect to untreated controls. The IC_50_ was defined as the drug concentration required to inhibit HCT116 or HCT116+ch3 by 50% relative to controls. IC_50_ values were estimated from the dose response curve. Data were derived from at least three independent experiments. This experiment was repeated 3 times independently, and the statistical analysis was done to obtain the final values.

### DAPI staining of apoptotic cells

To examine the apoptotic changes in HCT116 and HCT116+ch3 cells, DAPI (4′,6′-Diamidino-2-phenylindole, Hoechst 33258) nuclear staining assay was performed. For monolayer cultures 1×10^6^ cells/plate were seeded in 35-mm tissue culture discs. After 80–90% confluency, the cells were treated with different concentrations of curcumin or 5-FU (0, 1, 5, 10 and 20 µM) or a combination of curcumin (5 µM) and 5-FU (0.1, 1, 2 and 3 µM), calculated from the IC_50_ values, for 24 h. After completion of treatment the cells were fixed with methanol for 30 min at 4 °C in the dark. Fixed cells were washed twice with PBS, and then DAPI solution was spread over the plates followed by incubation for 1 h at 4 °C in the dark. Labeled cells were washed repeatedly with PBS to remove the excess DAPI stain and evaluated under fluorescence microscope (Leica, Germany).

### Transmission electron microscopy (TEM)

HCT116 and HCT116+ch3 colon cancer cells were treated with curcumin (20 µM), 5-FU (5 µM) or a combination of both (curcumin 5 µM and 5-FU 1 µM in HCT116, curcumin 5 µM and 5-FU 0.1 µM in HCT116+ch3) for 12, 24, 36, 48, 60 and 72 h, respectively, to determine the optimum time needed for inhibition of 50% cell growth. Electron microscopy was performed as previously described [30]. Briefly, cultures were fixed for 1 h in Karnovsky's fixative followed by post-fixation in 1% O_s_O_4_ solution. After dehydration in an ascending alcohol series, cultures were embedded in Epon and cut ultrathin with a Reichert-Jung Ultracut E (Darmstadt, Germany). Sections were contrasted with a mixture of 2% uranyl acetate/lead citrate and examined with a transmission electron microscope (Zeiss, Jena, Germany).

### Quantification of apoptotic cell death

Ultrathin sections of the samples were prepared and evaluated with an electron microscope (TEM 10; Zeiss). To quantify morphological evaluations and to define the time point at which 50% of the cells showed mitochondrial changes (MC) and/or were apoptotic, the number of cells with morphological features of apoptotic cell death including MC was determined by scoring 100 cells from 20 different microscopic fields.

### Cell Cycle Analysis by Flow Cytometry

HCT116 cells (1×10^6^) were plated in 60-mm tissue culture plates and after approximately 80% confluency cells were treated with 20 µM curcumin, 5 µM 5-FU, or their combination (5 µM curcumin and 1 µM 5-FU) for 12 and 24 h. In a separate experiment, HCT116+ch3 cells were treated with 5 µM curcumin, 1 µM 5-FU, or their combination (5 µM curcumin and 0.1 µM 5-FU) for 12 and 24 h. After treatment, cells were fixed using ice cold 70% ethanol, washed 2x with PBS and then resuspended with propidium iodide (10 µg/ml) and ribonuclease A (0.1%) in PBS for 30 min. Cells were incubated for 30 min in the dark at room temperature. Fluorescent events from propidium iodide–DNA complexes were quantified after laser excitation of the fluorescent dye by fluorescence-activated cell sorter (FACS) (Becton Dickinson, CA) with a cell count of 10,000 cells per sample. Finally, DNA content of the cells at different phases of the cell cycle was determined by using Cell Quest Software (Becton Dickinson). Experiments and analysis were performed in triplicate.

### Preparation of nuclear and cytoplasmic extracts to evaluate NF-κB translocation

The nuclear extracts were prepared as described previously [31]. Briefly, cells were suspended in 400 µl of hypotonic lysis buffer containing protease inhibitors for 20 min. The cells were then lysed with 12.5 µl of 10% Nonidet P-40. The homogenate was centrifuged for 1.5 minutes, and supernatant (cytoplasmic extracts) was stored frozen at −70 °C. Then 25 µl of ice-cold nuclear extraction buffer was added to the pellets and incubated for 30 minutes with intermittent mixing. Extracts were centrifuged and the supernatant (nuclear extracts) transferred to pre-chilled tubes for storage at −70 °C.

### Isolation of mitochondrial extracts for detection of cytochrome c-release

Cells were washed in 1 ml ice cold PBS and placed in ice-cold mitochondria isolation buffer (0.25 M sucrose, 0.2 mM EDTA, and 10 mM Tris–HCl, pH 7.8) for 30 min, followed by homogenization using a pre-cooled glass homogenizer. Cell lysates were centrifuged at 1000 g for 15 min at 4 °C and the supernatant was centrifuged at 12,000 g for 15 min at 4 °C. Isolated mitochondria were resuspended in mitochondria isolation buffer containing protease inhibitors (5 µg/ml leupeptin, 5 µg/ml pepstatin, 10 µg/ml aprotinin, 1 mM PMSF, 1 mM DTT, 100 mM sodium orthovanadate, 10 mM sodium fluoride, and 10 mM phenylarsine oxide).

### Western blot analysis

To determine the effects of curcumin, 5-FU or curcumin/5-FU on the HCT116 and HCT116+ch3 cells, whole cell lysates, cytoplasmic, mitochondrial and nuclear extracts of monolayer cultures were prepared and fractioned by SDS-PAGE [29], [32]. The total protein concentration of the cell extracts was determined using the bicinchoninic acid assay system (Uptima; Interchim, Montlucon, France) using BSA as a standard. Equal quantities (500 ng protein per lane) of total proteins were separated by SDS-PAGE (5%, 7.5%, 12% gels) under reducing conditions.

### Immune complex kinase assay

Immune complex kinase assay was performed as previously described in detail [33]. Briefly, to test the effect of curcumin and PI-3K inhibitor (wortmannin) on 5-FU-induced IKK activation, immune complex kinase assays were performed. The IKK complex was immunoprecipitated from whole cell lysates with antibodies against IKK-α and IKK-β and subsequently incubated with protein A/G-agarose beads (Pierce, Germany). After 2 h incubation, the beads were washed with lysis buffer and resuspended in a kinase assay solution containing 50 mM HEPES (pH 7.4), 20 mM MgCl2, 2 mM dithiothreitol, 10 µM unlabeled ATP and 2 mg of IKK substrate GST-IκBα (amino acid 1–54) and incubated at 30 °C for 30 min. This was followed by boiling in SDS-PAGE sample buffer for 5 min. Proteins were separated using SDS-page under reducing conditions as described above. Phosphorylation of GST-IκBα was assessed using a specific antibody against phospho-specific IκBα (Ser 32/36). To demonstrate the total amounts of IKK-α and IKK-β in each sample, whole-cell proteins were separated using SDS-PAGE under reducing conditions as described above. Detection of IKK-α and IKK-β was performed by immunoblotting with either anti-IKK-α or anti-IKK-β antibodies.

### Statistical analysis

Numerical data are expressed as mean values (+/−SD) for a representative experiment performed in triplicate. The means were compared using student's t-test assuming equal variances. Differences were considered to be statistically significant if the P-value was less than 0.05.

## Results

This study was designed to investigate how curcumin inhibits the proliferation of CRC cells and potentiates the effects of the chemotherapeutic agent 5-FU in an *in vitro* model of human CRC cells. Additionally, we examined the mechanism(s) by which curcumin enhances the anti-proliferative effects of 5-FU, especially concerning the effects on NF-κB and Src activation, NF-κB-regulated gene products, and cell growth in CRC cells. Two human CRC cells (HCT116 and HCT116+ch3) were used in this investigation.

### Curcumin sensitizes HCT116 and HCT116+ch3 colon cancer cells to 5-FU-treatment leading to decreased cell viability and proliferation

**Figure 1:**
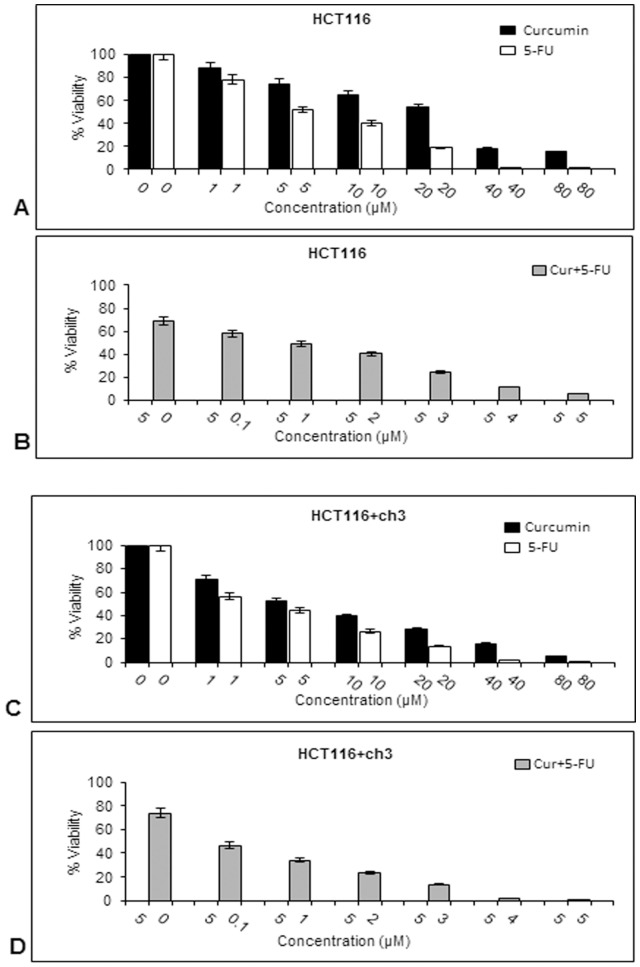
Effect of curcumin and/or 5-FU on cell viability and proliferation of HCT116 and HCT116+ch3 colon cancer cells. **A**: HCT116 cells were treated with different concentrations of curcumin or 5-FU (0, 1, 5, 10, 20, 40 and 80 µM) for 24 h and cell viability was measured using the MTT method. Concentrations of curcumin and 5-FU resulting in 50% growth inhibition were indicated as individual IC_50_ values. **B**: HCT116 cells were pretreated with curcumin (5 µM for 4 h), then exposed to 5-FU in different concentrations (0, 0.1, 1, 2, 3, 4 and 5 µM) for 24 h and evaluated by MTT assay. IC_50_ for 5-FU in combination treatment was determined at 50% growth inhibition of the HCT116 cells. The same experiments shown in (A) and (B) were performed on HCT116+ch3 cells. IC_50_ values for single (**C**) and combination treatment (**D**) were calculated on the basis of MTT measurements. The results are provided as mean values with standard deviations from at least three independent experiments. Values were compared to the control and statistically significant values with p<0.05.

The effects of 5-FU and/or curcumin on cell viability were evaluated by MTT assay in HCT116 and HCT116+ch3 cells (Fig. 1). On the basis of these measurements, the IC_50_ values (50% cell growth inhibitory concentrations) for the individual and combined drugs on HCT116 and HCT116+ch3 colon cancer cell viability were determined. The cells were exposed to different concentrations of curcumin or 5-FU (0, 1, 5, 10, 20, 40 and 80 µM) and cell viability was evaluated by MTT assay as described in Materials and Methods (Fig. 1A & 1C). The individual IC_50_ of curcumin and 5-FU were approximately 20 µM and 5 µM in HCT116 cells and 5 µM and 1 µM in HCT116+ch3 cells, respectively (*p<*0.05). These results suggest that curcumin and 5-FU have potent anti-proliferative effects in CRC cells and that these effects are more pronounced in HCT116+ch3 than in HCT116 cells.

To examine the effect of a combined treatment of curcumin and 5-FU, HCT116 or HCT116+ch3 cells were pretreated with 5 µM curcumin for 4 h and then co-treated with different concentrations of 5-FU (0, 0.1, 1, 2, 3, 4 and 5 µM) for 24 h (Fig. 1B & 1D). MTT assay was performed and IC_50_ values were determined. Interestingly, pretreatment with 5 µM curcumin reduced IC_50_ values for 5-FU to 1 µM in HCT116 and 0.1 µM in HCT116+ch3 (*p<*0.05) cells. These results indicate that cells pretreated with curcumin were more sensitive to 5-FU than cells treated with 5-FU alone and the introduction of chromosome 3 in HCT116 cells showed an increased sensitivity of the cells to the treatment with 5-FU and/or curcumin compared to the HCT116 wild type.

### The combination effect of curcumin and 5-FU on apoptosis in HCT116 and HCT116+ch3 cells

**Figure 2:**
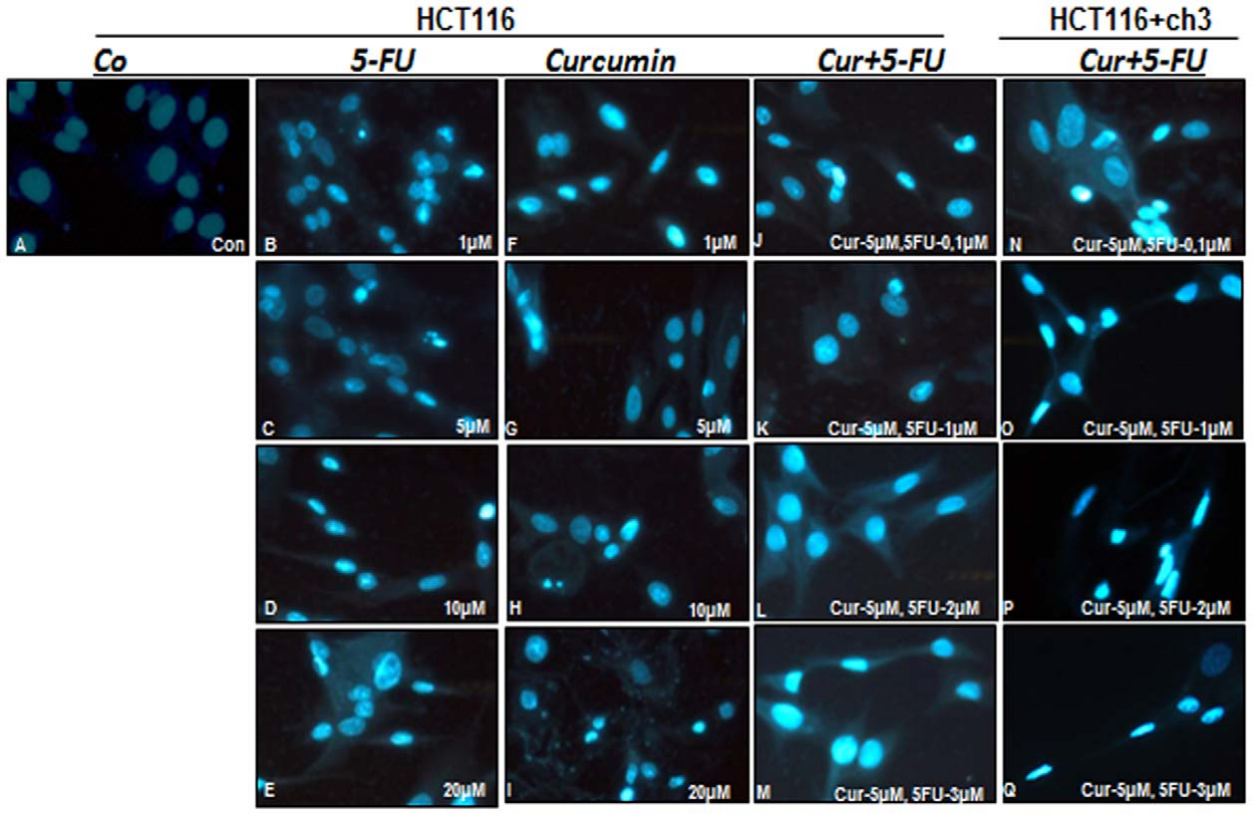
Effect of curcumin and/or 5-FU on apoptosis in HCT116 and HCT116+ch3 colon cancer cells. HCT116 and HCT116+ch3 cells were treated with different concentrations of curcumin or 5-FU (0, 1, 5, 10 and 20 µM) or a combination of curcumin (5 µM) and 5-FU (0.1, 1, 2 and 3 µM) for 24 h. Monolayer cultures were stained with Hoechst 33258 (DAPI) to reveal apoptotic changes in the cell nuclei.

To determine whether the inhibitory effect of curcumin and 5-FU on cell viability and cell growth is related to the induction of apoptosis, HCT116 and HCT116+ch3 cells were stained with Hoechst 33258 (DAPI). This fluorescence-based staining method reveals apoptotic bodies containing nuclear fragmentation and chromatin condensation in apoptotic cells. HCT116 and HCT116+ch3 cells were exposed to different concentrations of curcumin or 5-FU (0, 1, 5, 10 and 20 µM) or to a combination of curcumin (5 µM) and 5-FU (0.1, 1, 2 and 3 µM). Applied concentrations were calculated from the IC_50_-values of curcumin and 5-FU determined by MTT assays. As shown in Fig. 2, the number of apoptotic nuclei was markedly increased in cells of the combination treatment group. This confirmed the results in Fig. 1B & 1D and revealed that curcumin sensitizes HCT116 colon cancer cells to 5-FU-induced apoptosis. Furthermore, the restoration of hMLH1 activity in the HCT116 cells, by introduction of chromosome 3, was associated with an increased sensitivity to 5-FU and 5-FU-induced apoptosis.

### Curcumin enhances 5-FU-induced mitochondrial changes and apoptosis in HCT116 and HCT116+ch3 cells

**Figure 3:**
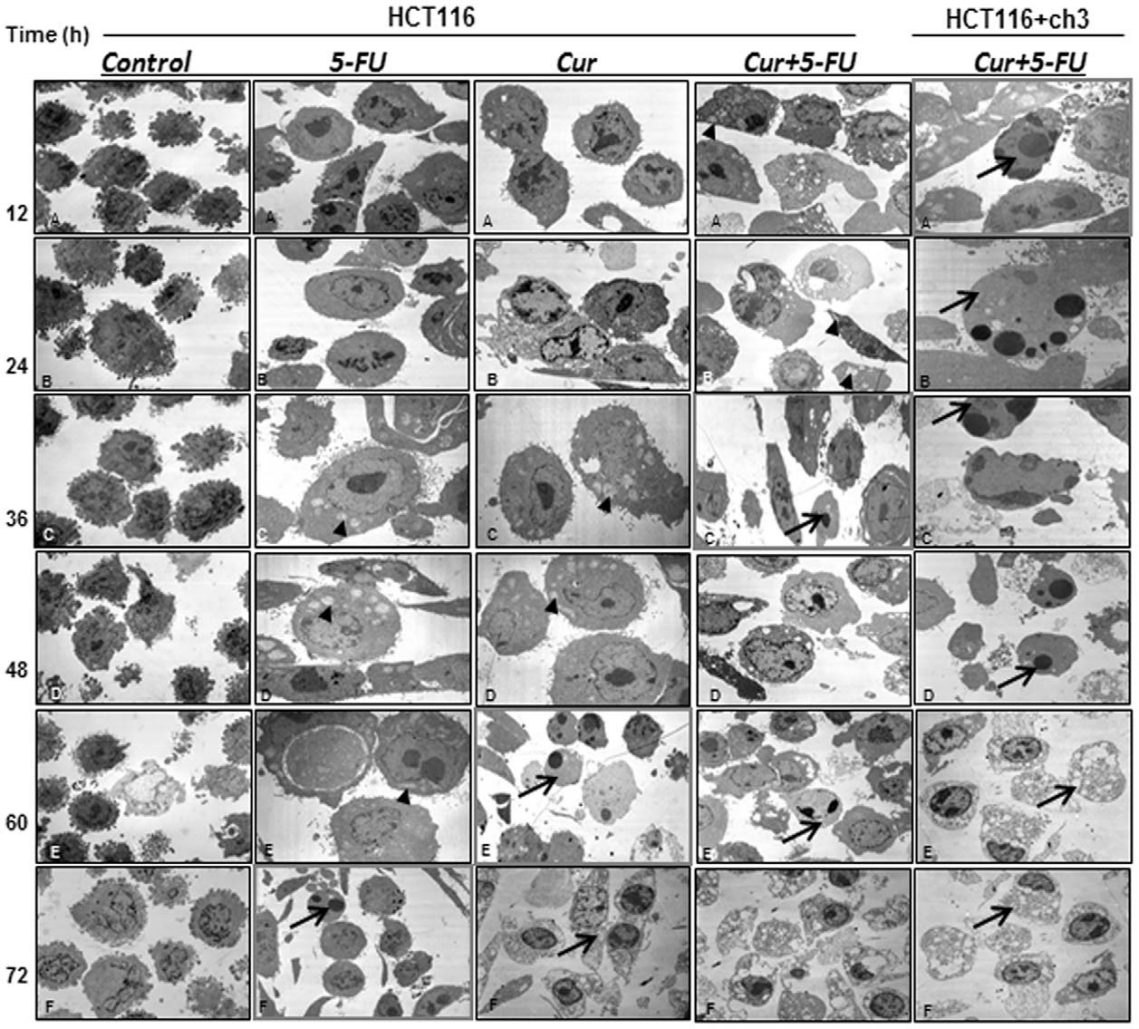
Ultrastructural evaluation of mitochondrial and apoptotic changes after treatment with curcumin and/or 5-FU in HCT116 and HCT116+ch3 colon cancer cells. HCT116 cells were treated with curcumin (20 µM), 5-FU (5 µM) or a combination of both (5 µM curcumin and 1 µM 5-FU) for 12, 24, 36, 48, 60 and 72 h. Using a different approach HCT116+ch3 cells were treated with a combination of 5 µM curcumin and 0.1 µM 5-FU for 12, 24, 36, 48, 60 and 72 h. Ultrathin sections were prepared and evaluated by transmission electron microscopy. Micrographs shown are representative of all the cultures evaluated. At the earliest time point when apoptosis was first detected images are highlighted arrows. Mitochondrial changes (arrowheads) are shown. Magnification: x5000, bar = 1 µm.

As shown in Fig. 3, HCT116 colon cancer cells were treated with curcumin (20 µM), 5-FU (5 µM) or a combination of both (curcumin 5 µM and 5-FU 1 µM) for 12, 24, 36, 48, 60 and 72 h, respectively. The viability and morphological changes of the cells were determined by ultrastructural examination with transmission electron microscopy. HCT116 colon cancer cells in control cultures exhibited a rounded/spherical morphology with small cytoplasmic processes, large nuclei (mostly euchromatic) with distinct nucleoli and a well-organized cytoplasm during the entire treatment duration (Fig. 3, Control:A–F). Treatment of HCT116 cultures with curcumin or 5-FU alone up to 36 hours resulted in degenerative changes, such as the appearance of multiple vacuoles, swelling of mitochondria and rough ER and degeneration of other cellular organelles (Fig. 3, 5-FU, Cur:A–C). Longer incubation periods with curcumin or 5-FU (60 and 72 hours) resulted in more cellular degeneration. This included areas of condensed heterochromatin in the cell nuclei and multiple, autophagic cytoplasmic vacuoles; the cells became apoptotic (Fig. 3, 5-FU:F, Cur:E–F). Pretreatment of HCT116 cultures with curcumin (5 µM) for 4 h, followed by co-treatment with 5-FU (1 µM) over the same time period revealed a strong effect. Extensive morphological degenerative features, mitochondrial swelling and apoptosis were found as early as 36 h in HCT116 cells and continued to accumulate until 72 h (Fig. 3, Cur+5-FU HCT116:C). In comparison to HCT116 cells, examinations were also performed on HCT116+ch3 cells treated with a combination of 5 µM curcumin and 0.1 µM 5-FU. Here, these effects were even more prominent and apoptotic cells were already detected after 12 hours of treatment (Fig. 3, Cur+5-FU HCT116+ch3:A). These results confirmed that sensitivity to 5-FU was enhanced by pretreatment with curcumin and that this was aggravated in HCT116+ch3 cells.

**Figure 4:**
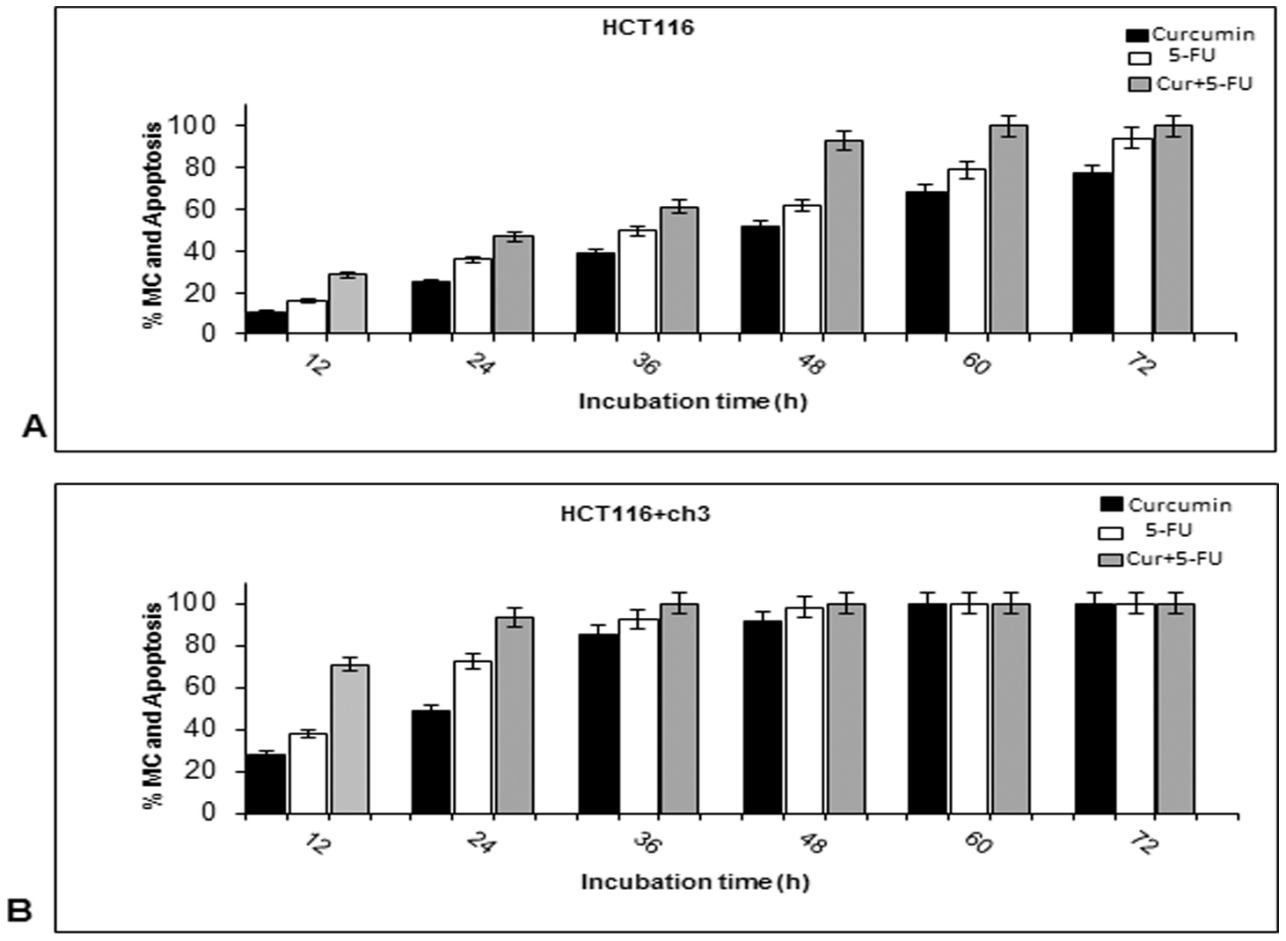
Quantification of mitochondrial and apoptotic changes after treatment with curcumin and/or 5-FU in HCT116 and HCT116+ch3 colon cancer cells. To quantify the ultrastructural findings, HCT116 (A) and HCT116+ch3 (B) cultures treated as described in Fig. 3 were examined for apoptotic and mitochondrial changes (MC) by counting 100 cells from 20 microscopic fields. The examination was performed in triplicate and the results are provided as mean values with standard deviations SD (*p*<0.05) from three independent experiments.

Quantification and statistical evaluation of the ultrastructural data clearly highlighted the time-dependent effects of curcumin and/or 5-FU treatment on mitochondrial changes (MC) and apoptosis in HCT116 and HCT116+ch3 cells. As shown in Fig. 4A & B, more than 50% of the cells exhibited MC or apoptotic features at 24 h incubation time in the combination experiments with HCT116 cells and at 12 h in the combination experiments with HCT116+ch3 cells (*p<*0.05). Again this suggests that curcumin sensitizes HCT116 and HCT116+ch3 cells to 5-FU-induced apoptosis. The results indicate that a very low quantity of 5-FU (0.1 µM) is required to suppress cell viability when combined to a moderate dose of curcumin (5 µM). Furthermore, these findings confirm that the introduction of chromosome 3 in HCT116 cells significantly increases sensitivity of the cells to the treatment with 5-FU and/or curcumin compared to the HCT116 cells.

### Effects of curcumin and/or 5-FU on cell cycle and apoptosis in HCT116 and HCT116+ch3 cells

**Figure 5:**
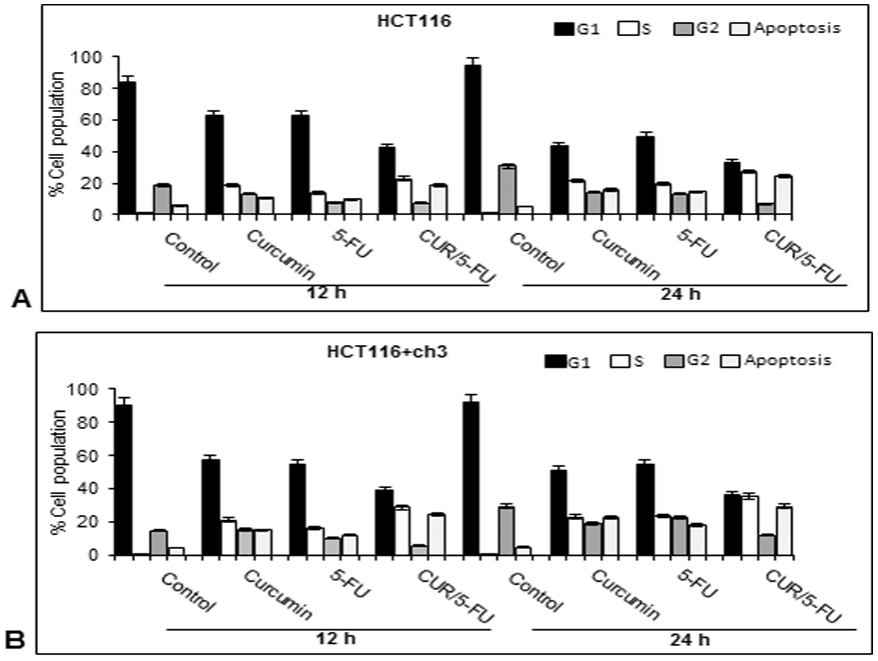
Effect of curcumin and/or 5-FU on the cell cycle of HCT116 and HCT116+ch3 colon cancer cells. HCT116 cells were treated with 20 µM curcumin or 5 µM 5-FU or a combination of 5 µM curcumin and 1 µM 5-FU for 12 and 24 h (**A**). HCT116+ch3 cells were treated with 5 µM curcumin or 1 µM 5-FU or a combination of 5 µM curcumin and 0.1 µM 5-FU for 12 and 24 h (**B**). Cell cycle analysis was performed by flow cytometry. These studies were performed in triplicate and the results presented are mean value with standard deviations from three independent experiments. Values are given as mean ± SD (*p*<0.05).

To examine the mechanisms by which curcumin and/or 5-FU inhibit the proliferation of HCT116 and HCT116+ch3 cells, we examined and compared their effect on the rate of growth inhibition and the levels of apoptosis. Therefore, we determined the behavior of these two CRC cells in the various phases of the cell cycle by flow cytometric analysis (Fig. 5A/B). HCT116 cells were treated with 20 µM curcumin or 5 µM 5-FU or their combination (5 µM curcumin and 1 µM 5-FU) for 12 and 24 h. In an independent experiment, HCT116+ch3 cells were treated with 5 µM curcumin or 1 µM 5-FU or their combination (5 µM curcumin and 0.1 µM 5-FU) for 12 and 24 h. Treated cells were harvested and processed for flow cytometric analysis. The most significant effect in both single and combined treatment was a time- and concentration-dependent reduction of cells in the G1-phase and accumulation of cells in the S-phase of the cell cycle. This effect was even more pronounced in HCT116+ch3 cells than in HCT116 cells. After 12 h treatment, which was the earliest time point we examined, the effects of curcumin and/or 5-FU on the cell cycle were already very distinct. Besides the changes in G1- and S-phase distribution, the number of cells undergoing apoptosis was markedly elevated with treatment. Interestingly, the effects of the combination treatment clearly exceeded those of the single treatments. At 24 h treatment, the influence on the cell cycle became overall more pronounced in both colon cancer cells, but was even more evident in HCT116+ch3 cells than in HCT116 cells. The results showed that the degree of cell growth inhibition and the levels of apoptosis were significantly higher in HCT116+ch3 cells than in HCT116 cells.

### Curcumin potentiates the antitumor activity of 5-FU through apoptotic signaling pathway in HCT116 and HCT116+ch3 cells

**Figure 6:**
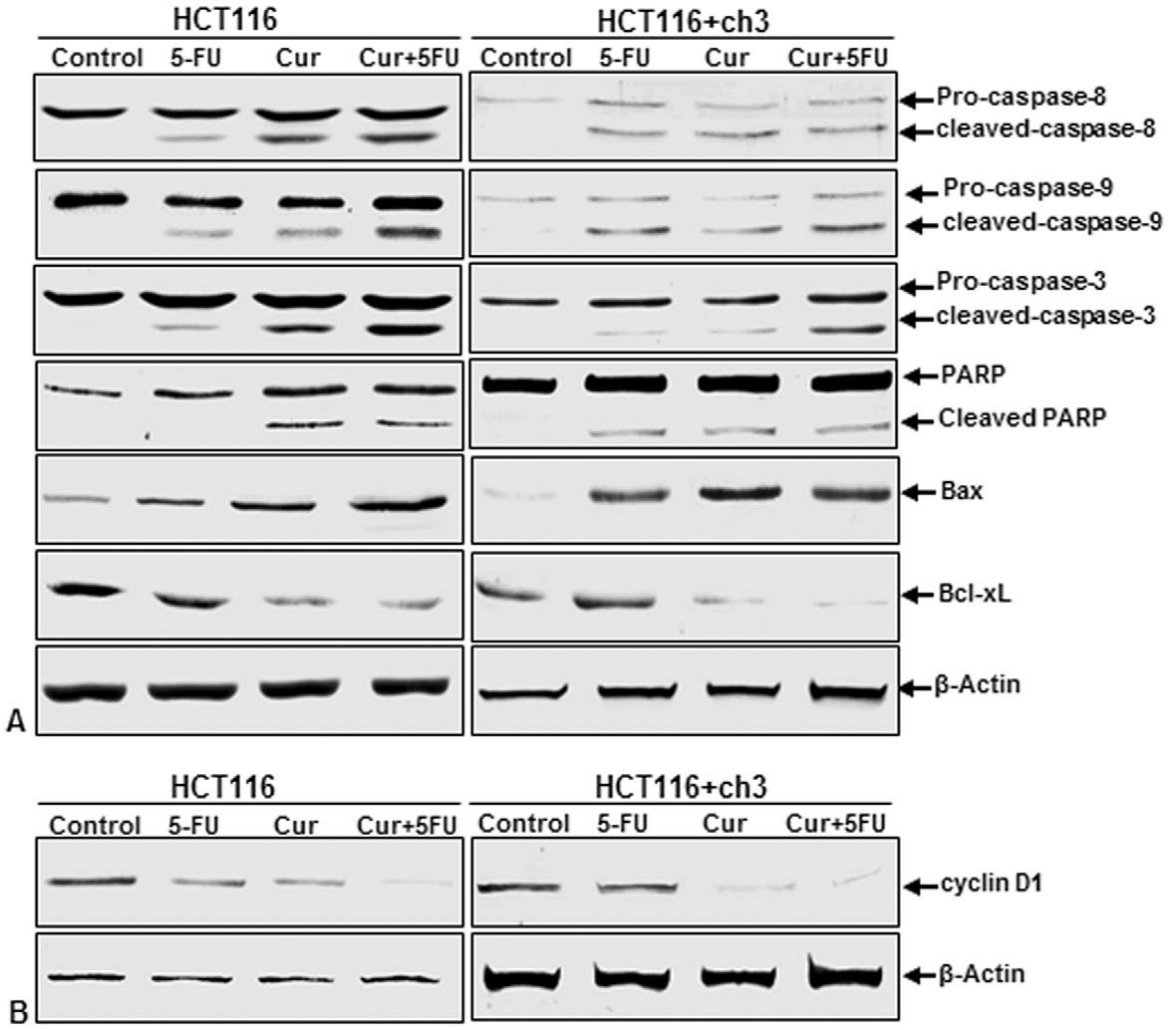
Effect of curcumin and/or 5-FU on apoptotic signaling in HCT116 and HCT116+ch3 colon cancer cells. HCT116 cells were treated with 20 µM curcumin or 5 µM 5-FU or a combination of 5 µM curcumin (4 h pretreatment) and 1 µM 5-FU for 24 h. HCT116+ch3 cells were treated with 5 µM curcumin or 1 µM 5-FU or a combination of 5 µM curcumin (4 h pretreatment) and 0.1 µM 5-FU for 24 h. Whole cell lysates were prepared and analyzed by western blotting for **A:** expression or cleavage of pro-apoptotic proteins caspase-8, caspase-9, caspase-3, PARP and Bax, and of anti-apoptotic protein BCL-xL **B:** expression of cyclin D1. The housekeeping protein β-actin served as a positive loading control in all experiments.

Activation of caspases induces apoptosis in cells. Furthermore, activation of caspase-8 (extrinsic pathway) and -9 (intrinsic pathway) stimulates caspase-3, which in turn induces PARP cleavage [34]. Therefore, we examined whether curcumin and/or 5-FU can modulate the expression or cleavage of pro-apoptotic proteins such as caspase-8, caspase-9, caspase-3, Bax, and PARP or of anti-apoptotic proteins such as BCL-xL in HCT116 and HCT116+ch3 cells. As shown in Fig. 6A, immunoblot analysis showed that the expression/cleavage of caspase-8, caspase-9, caspase-3, Bax, and PARP was enhanced, while the expression of BCL-xL was decreased when HCT116 cells were exposed to curcumin (20 µM) or 5-FU (5 µM) and HCT116+ch3 cells were exposed to curcumin (5 µM) or 5-FU (1 µM) as single agents, or in the combination of curcumin and 5-FU (5 µM+1 µM: HCT116 cells, 5 µM+0.1 µM: HCT116+ch3 cells, respectively). It is important to note that these effects were more pronounced in combination treatment with curcumin and 5-FU than in single treatments. These results indicate that pretreatment with curcumin sensitizes the cells to 5-FU-induced apoptosis in HCT116 and HCT116+ch3 cells by both the extrinsic and intrinsic pathways.

### Curcumin enhances the antitumor activity of 5-FU through down-regulation of proteins associated with proliferation in HCT116 and HCT116+ch3 cells

We examined whether suppression of proliferation of HCT116 and HCT116+ch3 cells by curcumin or/and 5-FU is due to down-regulation of proteins involved in cell proliferation, such as cyclin D1. Curcumin or/and 5-FU blocked expression of cyclin D1 in HCT116 cells and HCT116+ch3 (Fig. 6B). This result suggests that pretreatment with curcumin sensitizes the cells to 5-FU-induced cyclin D1 inactivation, most likely through the suppression of cell proliferation proteins.

### Curcumin potentiates 5-FU-induced cytochrome c release in HCT116 and HCT116+ch3 cells

**Figure 7:**
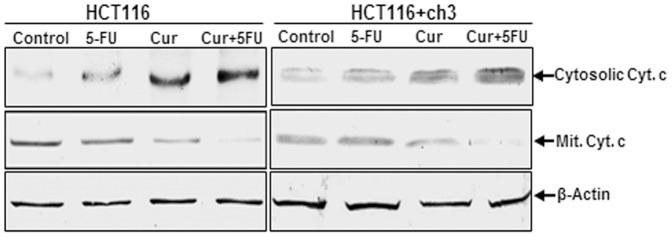
Effect of curcumin and/or 5-FU on mitochondrial damage and cytochrome c release in HCT116 and HCT116+ch3 colon cancer cells. HCT116 cells were treated with 20 µM curcumin or 5 µM 5-FU or a combination of 5 µM curcumin (4 h pretreatment) and 1 µM 5-FU for 24 h. HCT116+ch3 cells were treated with 5 µM curcumin or 1 µM 5-FU or a combination of 5 µM curcumin (4 h pretreatment) and 0.1 µM 5-FU for 24 h. Mitochondrial and cytoplasmic cell fractions were prepared and analyzed by western blotting using antibodies against cytochrome c. The housekeeping protein β-actin served as a loading control.

Activation of caspase-8 induces cytochrome *c* release from mitochondria, which then activates caspase-9 and -3 [34]. Therefore, we examined whether pretreatment with curcumin not only potentiates 5-FU-induced caspase-activation, but also leads to enhanced cytochrome c release in HCT116 and HCT116+ch3 cells. Cells were treated with 5-FU or curcumin alone or pretreated with curcumin and then treated with 5-FU as described above. Mitochondrial and cytosolic fractions from HCT116 and HCT116+ch3 cells were prepared and examined for cytochrome c release by western blot analysis (Fig. 7). The cytosolic cytochrome c increased in the presence of 5-FU or curcumin and even more in the combination treatment with both agents. At the same time, there was a corresponding decrease in the mitochondrial compartment. Thus, these results suggest that pretreatment with curcumin sensitizes the cells to 5-FU-induced apoptosis (Fig. 6A) most likely through the release of cytochrome c from mitochondria.

### Curcumin suppresses 5-FU-induced nuclear factor-κB and PI-3K/Src in HCT116 and HCT116+ch3 cells

**Figure 8:**
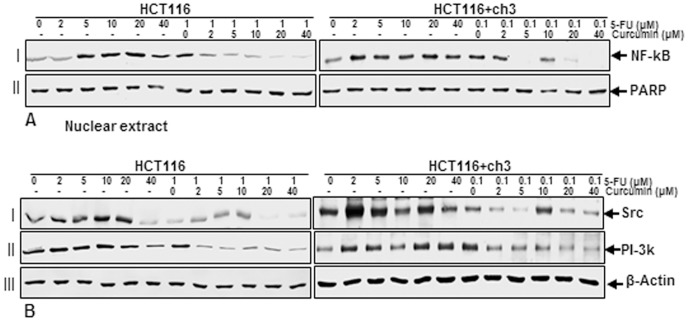
Effect of curcumin and/or 5-FU on NF-κB and PI-3K/Src activation in HCT116 and HCT116+ch3 colon cancer cells. HCT116 and HCT116+ch3 cells were either treated with different concentrations of 5-FU (0, 2, 5, 10, 20, 40 µM) alone for 1 h or were pretreated with different concentrations of curcumin (0, 2, 5, 10, 20, 40 µM) for 1 h and then exposed to 1 µM (HCT116) or 0.1 µM (HCT116+ch3) 5-FU for 1 h. **A:** After preparation of nuclear extracts western blotting was performed with antibodies against NF-κB and PARP as a loading control. **B:** Cytoplasmic fractions were subsequently examined by western blotting for expression of PI-3K (lane I), Src (lane II) and β-actin (lane III) (loading control).

Previous papers from our group and other laboratories focusing on signaling pathways suggest that PI-3K signaling is involved in cytokine-stimulated nuclear factor-κB (NF-κB) activation [32], [35]. Furthermore, transcription factor NF-κB and PI-3K signaling pathways have been reported to be associated with cell proliferation, invasion, angiogenesis, metastasis, anti-apoptosis and chemoresistance in multiple tumor cells [36], [37]. To examine whether NF-κB and PI-3K activity is involved in the 5-FU stimulated HCT116 and HCT116+ch3 cells and whether curcumin suppresses NF-κB and PI-3K activation, HCT116 and HCT116+ch3 cells were either treated with 5-FU (0, 2, 5, 10, 20, 40 µM) for 1 h or were pretreated with curcumin (0, 2, 5, 10, 20, 40 µM) for 1 h and then co-treated with 5-FU (1 µM: HCT116, 0.1 µM: HCT116+ch3) for 1 h. As shown in Fig. 8 A/B, 5-FU stimulated the expression of NF-κB (Fig. 8A, lane I) and PI-3K/Src (Fig. 8B, lane I and II) in HCT116 and HCT116+ch3 cells in a dose-dependent manner and curcumin suppressed this 5-FU-induced NF-κB and PI-3K/Src activation. Taken together, these results support a functional role for the PI-3kinase pathway in 5-FU-induced signaling pathway.

### Suppression of 5-FU-induced activation of IKK by curcumin might involve PI-3K signaling pathway in HCT116 and HCT116+ch3 cells

**Figure 9:**
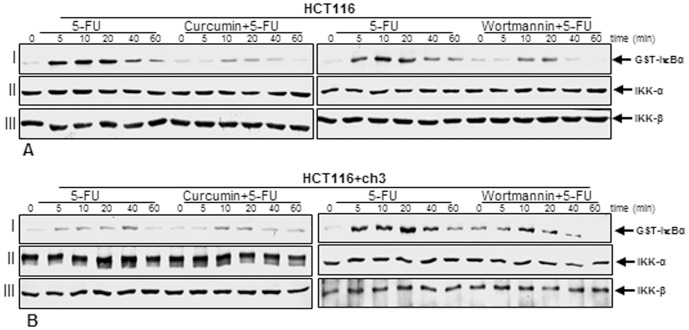
Effect of 5-FU and/or curcumin or PI-3K inhibitor wortmannin on activation of IκBα kinase (IKK) in HCT116 and HCT116+ch3 colon cancer cells. A: HCT116 cells were treated with 5-FU (5 µM) for 0, 5, 10, 20, 40, or 60 minutes or were pretreated with curcumin (5 µM) or wortmannin (10 nM) for 1 h and then co-treated with 1 µM 5-FU for 0, 5, 10, 20, 40, or 60 minutes. B: HCT116+ch3 cells were treated with 5-FU (1 µM) for 0, 5, 10, 20, 40, or 60 minutes or were pretreated with curcumin (5 µM) or wortmannin (10 nM) for 1 h and then co-treated with 0.1 µM 5-FU for 0, 5, 10, 20, 40, or 60 minutes. Cells were lysed and immune complex kinase assays were performed as described in Materials and Methods. Equal amounts of total protein (500 ng protein per lane) were separated by SDS-PAGE under reducing conditions and then analyzed by immunoblotting using antibodies against phosphospecific IκBα (lane I), IKK-α (lane II), and IKK-β (lane III).

The activated NF-κB subunit p65 translocates to the nucleus after phosphorylation, ubiquitination, and proteolytic degradation of IκBα [6], [38]. To identify the role of PI-3K in 5-FU-induced signaling and the mechanisms of curcumin's inhibitory effect on 5-FU-induced NF-κB transcriptional activity, we used a specific PI-3K inhibitor (wortmannin) in comparison to curcumin to see whether both agents blocked 5-FU-induced IKK activation in a similar manner. HCT116 (Fig. 9A) and HCT116+ch3 (Fig. 9B) cells were either treated with 5 µM (A), 1 µM (B) 5-FU alone for 0, 5, 10, 20, 40, or 60 minutes or were pretreated with curcumin (5 µM) or wortmannin (10 nM) for 1 h and then co-treated with 1 µM (A), 0.1 µM (B) 5-FU for the indicated times. The results from the immune complex kinase assay, performed as described in Materials and Methods, showed that 5-FU induced the activation of IKK in a time-dependent manner, while pretreatment of cells with curcumin or PI-3K inhibitor wortmannin followed by stimulation with 5-FU blocked the 5-FU-induced effects on the activation of IKK to a similar extent (Fig. 9A/B, *lane I*). This suggests that curcumin-mediated down-regulation of the 5-FU-induced NF-κB activation in CRC cells, could involve, at least in part, inhibition of the PI-3K signaling pathway. 5-FU, curcumin or wortmannin had no direct effect on the expression of IKK-α or IKK-β proteins (Fig. 9A/B, *lanes II* and *III*).

## Discussion

The incidence of CRC and its mortality rate are steadily increasing. Currently available chemotherapeutic agents for the treatment of CRC are associated with numerous side effects, development of treatment resistance and, until now, have not improved patient survival. Therefore, non-toxic and more selective pharmacotherapies are needed for CRC. Combination treatments may enhance the responses of CRC cells to chemotherapeutic agents.

Epidemiological studies suggest that plant polyphenols have the potential to sensitize tumor cells to chemotherapy by specifically suppressing signaling pathways that are involved in the development of treatment resistance induced by conventional chemotherapeutic drugs. How these polyphenols protect and display proliferative effects in healthy cells while sensitizing tumor cells and making them more susceptible to chemotherapeutics is not fully understood. The majority of chemotherapeutic agents stimulate transcription factor NF-κB, which mediates survival, proliferation, invasion and metastasis of cancer cells. Curcumin is a naturally occurring yellow pigment present in the spice turmeric, which is a potent inhibitor of NF-κB activation. Several studies have shown that the phytochemical curcumin displays anti-tumor effects in various solid tumors and potentiates the effect of a number of chemotherapeutic agents leading to increased sensitivity even in drug-resistant cancer cells [25], [39], [40]. The chemotherapeutic agent 5-FU is routinely used as a standard therapy for treatment of CRC, but with limited success [41]. In this study we hypothesized that curcumin might potentiate the anti-proliferative effect of chemotherapeutic agents in advanced CRC and inhibit metastasis. Therefore the primary goal of this study was to determine whether curcumin can sensitize CRC cells to 5-FU and to investigate the mechanism of action of this chemosensitization. For modeling CRC cells we used HCT116 (wild type) and HCT116+ch3 cells (modified by transfer of chromosome 3). The treatment with curcumin and 5-FU caused significantly more anti-proliferative effects and apoptosis in HCT116+ch3 and HCT116 cells compared to the individual drugs.

This study demonstrates that combining curcumin and 5-FU leads to a significant suppression of growth and viability of CRC cells, indicating that curcumin sensitizes the 5-FU surviving CRC cells to treatment. This is consistent with the observations of Yu and colleagues that curcumin is highly effective in sensitizing the FOLFOX surviving CRC cells [42].

The HCT116+ch3 cells were more sensitive to the treatment with curcumin and/or 5-FU than the HCT116 wild type cells. Therefore, there might be a mechanistic relationship between the combined drug action and DNA damage repair pathway. The deficiency of the mismatch repair (MMR) system in tumor cells could be important for clinical resistance to chemotherapy and this should be carefully considered for the selection and use of the therapeutic agents. This study employed the mismatch repair defective human colon carcinoma cell line HCT116 which has a mutation in the *hMLH1* gene, and a cell line where hMLH1 expression was restored by chromosome 3 transfer (HCT116+ch3). The increased sensitivity of HCT116+ch3 cells to the treatment with 5-FU and/or curcumin compared to HCT116 cells, suggests that the two cell populations respond differentially to 5-FU. This is consistent with the observations of Sargent et al. that CRC patients with defective DNA mismatch repair (III stage) do not benefit from 5-FU treatment [43], proposing that different mismatch CRCs could exhibit different chemosensitivity patterns.

Cell cycle analysis showed that untreated HCT116 and HCT116+ch3 cells underwent normal cell cycle with a transitory S phase and a high amount of cells in G1 phase. In contrast, the cells accumulated in the S phase and subsequently became apoptotic, instead of proceeding from S to G2 phase, after treatment with 5-FU anti-cancer drugs or/and curcumin. The S phase of the cell cycle is connected with the major cellular event of replication [44]. The combination of curcumin and 5-FU caused increased apoptosis in CRC cells compared to treatment with the single agents. There might be a connection between the combined treatment with 5-FU or/and curcumin and DNA damage repair pathway. Additionally, the decrease in cell growth and the degree of apoptotic cell death were significantly higher in HCT116+ch3 cells compared to HCT116 cells.

Curcumin also affected NF-κB–regulated gene products involved in apoptosis (caspase-3, -8, -9, PARP, Bax), anti-apoptosis (Bcl-xL) and proliferation (cyclin D1), thereby suppressing cell proliferation and potentiating 5-FU-induced apoptosis in CRC cells. While 5-FU activated NF-κB/PI-3K/Src pathway, which could provide a pro-survival response to chemotherapeutic agents and may account for the development of chemoresistance, curcumin down-regulated these signaling pathways and through this potentiated the anti-tumor effects of chemotherapy. Stimulation of tumor cells with anti-tumor drugs, cytokines and radiation can induce the activation of NF-κB and these cells can develop resistance to apoptosis and cytotoxicity induced by chemotherapy or radiotherapy by expression of anti-apoptotic genes [11], [45], [46], [47], [48]. The data presented suggest that HCT116 cells exhibit a higher susceptibility to the combination therapy than to single agents, suggesting that this treatment may be an effective therapeutic strategy for targeting chemoresistant cancer cells. Moreover, the combination of phytochemicals with standard chemotherapy can significantly reduce the dosage of the chemotherapeutic agents and thereby minimize adverse side effects and drug toxicity for the patients. Curcumin is a well-tolerated phytochemical, which could prevent primary tumor formation or tumor recurrence. Here, we provide evidence that curcumin may have chemopreventive potential against CRC by affecting multiple cell signaling molecules. Furthermore, we found that this compound suppressed activation of NF-κB and PI-3K/Src signaling pathways. The development of resistance to chemotherapeutic agents is a common phenomenon in tumor cells leading to recurrence of the tumor and several lines of evidence have reported that NF-κB and PI-3K signal transduction pathways are involved in the development of resistance of tumors against a numerous of anticancer chemotherapeutic agents [49], [50], [51].

The study also demonstrates that inhibition of the PI-3K signaling pathway enhances 5-FU-induced apoptosis in HCT116 cells. The release of mitochondrial mediators of apoptosis such as cytochrome c and apoptosis inducing factor is associated with mitochondrial damage. Activated Akt, a downstream kinase of PI-3K can phosphorylate BAD, a pro-apoptotic protein, and BAD is able to interact with Bcl-2 or Bcl-XL resulting in suppression of apoptosis [52]. Consistent with its function in the PI-3K signaling cascade, the inhibition of PI-3K by curcumin and subsequent blockage of Akt may induce apoptosis. The tyrosine kinase Src, that regulates cellular signaling pathways, has been linked with different stages of tumor progression such as CRC metastasis [53], [54] and implicated in treatment resistance to chemotherapeutic agents [55]. Indeed, it has been reported that suppression of Src signaling sensitizes tumor cells to chemotherapies [56], [57]. In agreement with these findings, we clearly demonstrate that curcumin alone or in combination with 5-FU markedly decreases Src expression in a dose-dependent manner in HCT116 cells, suggesting the inhibition of Src activity by curcumin imparts higher sensitivity to 5-FU.

**Figure 10:**
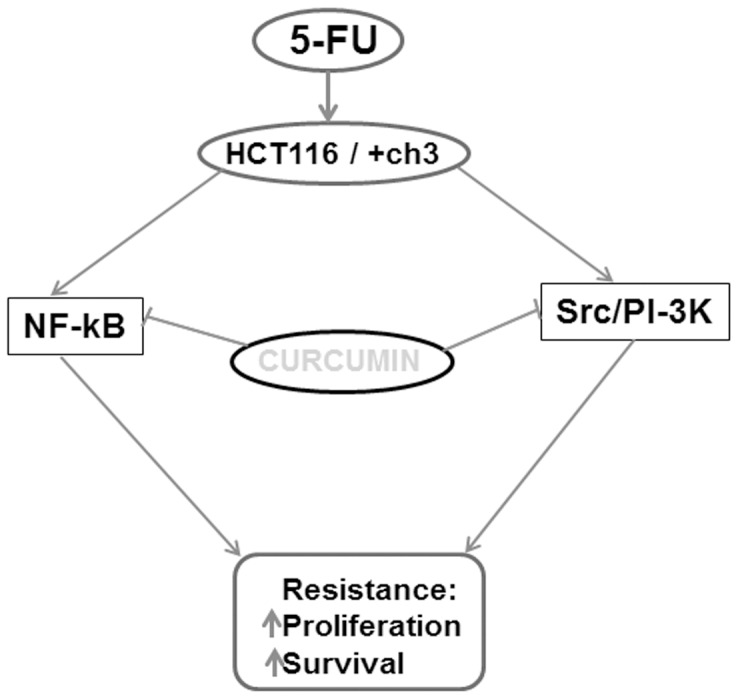
Schematic diagram illustrating the signaling pathways involved in the development of chemotherapeutic treatment resistance to 5-FU and chemosensitization by curcumin in HCT116 and HCT116+ch3 colon cancer cells.

In conclusion, the data presented in this study demonstrate that a pro-survival, anti-apoptotic signaling response of CRC cells to 5-FU is mediated via the NF-κB and PI-3K signaling pathways and curcumin modulates this response by targeting these inducible signaling pathways (Fig. 10). Moreover, we have shown that HCT116+ch3 cells are more sensitive to 5-FU and/or curcumin than HCT116 cells, suggesting an important role for the *hMLH1* gene in DNA repair and drug sensitivity. Overall, our results indicate that the combination of curcumin, which is a pharmacologically safe natural compound, with conventional chemotherapeutics like 5-FU could provide an improved strategy for colon cancer therapy.
