# Expression of Concern: Curcumin Enhances the Effect of Chemotherapy against Colorectal Cancer Cells by Inhibition of NF-κB and Src Protein Kinase Signaling Pathways

**DOI:** 10.1371/journal.pone.0314637

**Published:** 2024-11-22

**Authors:** The PLOS ONE Editors

After this article [1] was published, concerns were raised about Fig 3 and Fig 6.

Specifically:

- In Fig 3:
  ○ In the HCT116+ch3 cells, panel E (60h) appears similar to panel F (72h), when rotated 180 degrees.
  ○ Panel D (48h) from the HCT116+ch3 cells appears similar to panel e (40h) from the HCT116WT cells in Fig 4A of [2].
  ○ Panel C (36h) from the HCT116+ch3 cells appears similar to panel d (20h) from the HCT116WT cells in Fig 4A of [2].
- In Fig 6A, there appear to be horizontal background discontinuities in lanes 3 and 4 of the pro-caspase-8/cleaved-caspase-8 HCT116+ch3 panel when levels are adjusted.

The corresponding author acknowledged the above concerns but stated that the original data are no longer available. In the absence of the raw data, the issues with these figures cannot be resolved.

In light of the above concerns, the *PLOS ONE* Editors issue this Expression of Concern.
